# Diaqua­bis­(4-formyl­benzoato-κ*O* ^1^)bis­(nicotinamide-κ*N* ^1^)zinc

**DOI:** 10.1107/S160053681203320X

**Published:** 2012-07-28

**Authors:** Mustafa Sertçelik, Nagihan Çaylak Delibaş, Hacali Necefoğlu, Tuncer Hökelek

**Affiliations:** a Department of Chemistry, Kafkas University, 36100 Kars, Turkey; b Department of Physics, Sakarya University, 54187 Esentepe, Sakarya, Turkey; c Department of Physics, Hacettepe University, 06800 Beytepe, Ankara, Turkey

## Abstract

In the title complex, [Zn(C_8_H_5_O_3_)_2_(C_6_H_6_N_2_O)_2_(H_2_O)_2_], the Zn^II^ cation is located on an inversion center and is coordinated by two 4-formyl­benzoate (FB) anions, two nicotinamide (NA) ligands and two water mol­ecules. The four O atoms in the equatorial plane around the Zn^II^ cation form a slightly distorted square-planar arrangement, while the slightly distorted octa­hedral coordination is completed by the two N atoms of the NA ligands in the axial positions. The dihedral angle between the carboxyl­ate group and the adjacent benzene ring is 24.13 (8)°, while the pyridine ring and the benzene ring are oriented at a dihedral angle of 88.52 (4)°. The coordinating water mol­ecule links with the carboxyl­ate group *via* an O—H⋯O hydrogen bond. In the crystal, N—H⋯O and O—H⋯O hydrogen bonds, and a weak C—H⋯π inter­action link the mol­ecules into a two-dimensional network parallel to (010). These networks are linked *via* C—H⋯O and π–π inter­actions between inversion-related benzene rings [centroid–centroid distance = 3.8483 (7) Å], forming a three-dimensional supra­molecular structure.

## Related literature

For literature on niacin, see: Krishnamachari (1974 ▶). For information on the nicotinic acid derivative *N*,*N*-diethyl­nicotinamide, see: Bigoli *et al.* (1972 ▶). For related structures, see: Aydın *et al.* (2012 ▶); Hökelek *et al.* (2009 ▶); Necefoğlu *et al.* (2011 ▶); Sertçelik *et al.* (2012*a* ▶,*b* ▶,*c* ▶,*d* ▶); Sertçelik *et al.* (2009*a* ▶,*b* ▶,*c* ▶). For bond-length data, see: Allen *et al.* (1987 ▶). 
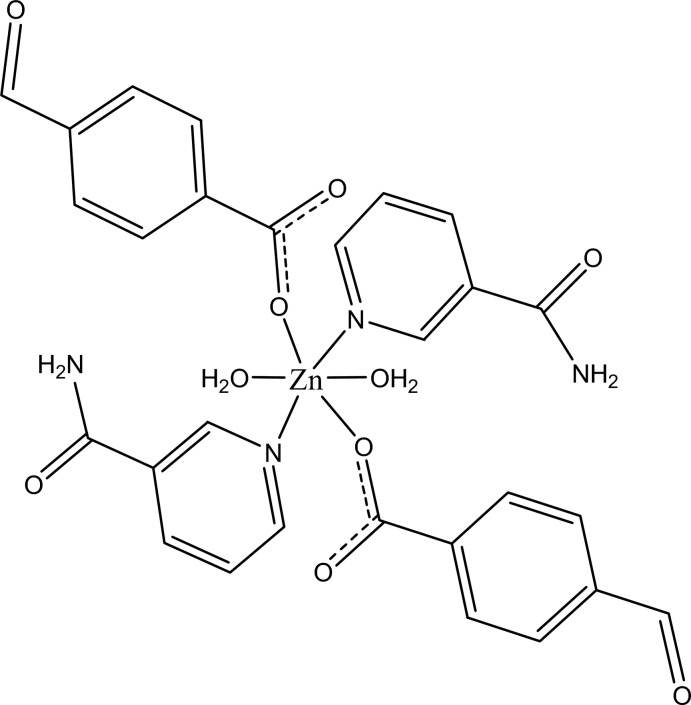


## Experimental

### 

#### Crystal data

[Zn(C_8_H_5_O_3_)_2_(C_6_H_6_N_2_O)_2_(H_2_O)_2_]
*M* *_r_* = 643.92
Triclinic, 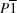

*a* = 7.7861 (2) Å
*b* = 9.7877 (3) Å
*c* = 9.9087 (3) Å
α = 77.851 (3)°
β = 71.462 (2)°
γ = 86.720 (3)°
*V* = 699.87 (4) Å^3^
*Z* = 1
Mo *K*α radiation
μ = 0.94 mm^−1^
*T* = 100 K
0.44 × 0.37 × 0.20 mm

#### Data collection

Bruker Kappa APEXII CCD area-detector diffractometer
Absorption correction: multi-scan (*SADABS*; Bruker, 2005 ▶) *T*_min_ = 0.682, *T*_max_ = 0.831
12257 measured reflections
3475 independent reflections
3413 reflections with *I* > 2σ(*I*)
*R*_int_ = 0.019

#### Refinement

*R*[*F* ^2^ > 2σ(*F* ^2^)] = 0.022
*wR*(*F* ^2^) = 0.060
*S* = 1.06
3475 reflections
216 parameters
H atoms treated by a mixture of independent and constrained refinement
Δρ_max_ = 0.43 e Å^−3^
Δρ_min_ = −0.31 e Å^−3^

### 

Data collection: *APEX2* (Bruker, 2007 ▶); cell refinement: *SAINT* (Bruker, 2007 ▶); data reduction: *SAINT*; program(s) used to solve structure: *SHELXS97* (Sheldrick, 2008 ▶); program(s) used to refine structure: *SHELXL97* (Sheldrick, 2008 ▶); molecular graphics: *ORTEP-3 for Windows* (Farrugia, 1997 ▶); software used to prepare material for publication: *WinGX* (Farrugia, 1999 ▶) and *PLATON* (Spek, 2009 ▶).

## Supplementary Material

Crystal structure: contains datablock(s) I, global. DOI: 10.1107/S160053681203320X/su2483sup1.cif

Structure factors: contains datablock(s) I. DOI: 10.1107/S160053681203320X/su2483Isup2.hkl

Additional supplementary materials:  crystallographic information; 3D view; checkCIF report

## Figures and Tables

**Table 1:** Hydrogen-bond geometry (Å, °) *Cg*2 is the centroid of the pyridine ring.

*D*—H⋯*A*	*D*—H	H⋯*A*	*D*⋯*A*	*D*—H⋯*A*
N2—H21⋯O2^i^	0.862 (17)	2.087 (17)	2.8789 (13)	152.4 (16)
N2—H22⋯O4^ii^	0.849 (17)	2.058 (18)	2.8904 (15)	166.4 (18)
O5—H51⋯O4^iii^	0.79 (2)	2.10 (2)	2.8597 (13)	161 (2)
O5—H52⋯O2^iv^	0.86 (2)	1.85 (2)	2.6845 (13)	163 (2)
C4—H4⋯O2^iii^	0.93	2.40	3.3245 (16)	173
C13—H13⋯O3^v^	0.93	2.47	3.3083 (17)	150
C6—H6⋯*Cg*2^vi^	0.93	2.72	3.6361 (14)	167

Symmetry codes: (i) 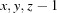; (ii) 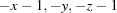; (iii) 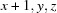; (iv) 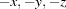; (v) 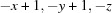; (vi) 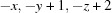.

**Fig. 1.**
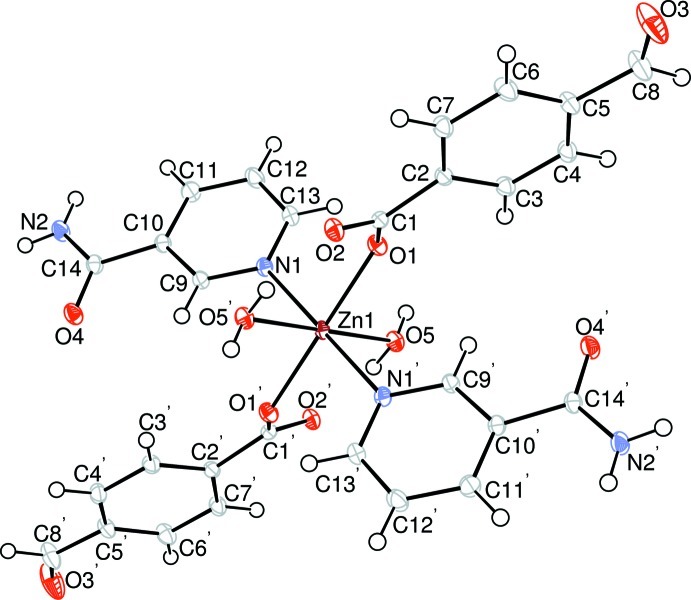
The molecular structure of the title molecule with the atom-numbering. Displacement ellipsoids are drawn at the 50% probability level [symmetry code: (') -x, -y, -z].

## supplementary crystallographic information

### Comment

As a part of our ongoing investigations of transition metal complexes of nicotinamide (NA), one form of niacin (Krishnamachari, 1974), and/or the nicotinic acid derivative *N*,*N*-diethylnicotinamide (DENA), an important respiratory stimulant (Bigoli *et al.*, 1972), the title compound was synthesized and its crystal structure is reported on herein.

In the title mononuclear complex, Zn^II^ cation is located on an inversion center and is coordinated by two 4-formylbenzoate (FB) anions, two nicotinamide (NA) ligands and two water molecules, all ligands coordinating in a monodentate manner (Fig. 1). The crystal structures of similar complexes of Cu^II^, Co^II^, Ni^II^, Mn^II^ and Zn^II^ ions, [Cu(C_8_H_5_O_3_)_2_(C_6_H_6_N_2_O)_2_(H_2_O)_2_] (Sertçelik *et al.*, 2012*a*), [Cu(C_7_H_4_BrO_2_)_2_(C_6_H_6_N_2_O)_2_(H_2_O)_2_] (Necefoğlu *et al.*, 2011), [Co(C_8_H_5_O_3_)_2_(C_10_H_14_N_2_O)_2_(H_2_O)_2_] (Sertçelik *et al.*, 2009*a*), [Co(C_8_H_5_O_3_)_2_(C_6_H_6_N_2_O)_2_(H_2_O)_2_] (Sertçelik *et al.*, 2012*b*), [Co(C_7_H_4_IO_2_)_2_(C_6_H_6_N_2_O)_2_(H_2_O)_2_] (Aydın *et al.*, 2012), [Ni(C_8_H_5_O_3_)_2_(C_10_H_14_N_2_O)_2_(H_2_O)_2_] (Sertçelik *et al.*, 2009*b*), [Ni(C_8_H_5_O_3_)_2_(C_6_H_6_N_2_O)_2_(H_2_O)_2_] (Sertçelik *et al.*, 2012*c*), [Mn(C_8_H_5_O_3_)_2_(C_10_H_14_N_2_O)_2_(H_2_O)_2_] (Sertçelik *et al.*, 2009*c*), [Zn(C_7_H_4_BrO_2_)_2_(C_6_H_6_N_2_O)_2_(H_2_O)_2_] (Hökelek *et al.*, 2009) and [Zn(C_8_H_5_O_3_)_2_(C_10_H_14_N_2_O)_2_(H_2_O)_2_] (Sertçelik *et al.*, 2012*d*) have also been reported, where all the ligands coordinate to the metal atoms in a monodentate manner.

In the title complex, the four symmetry related O atoms (O1, O1', O5 and O5') in the equatorial plane around the Zn^II^ ion form a slightly distorted square-planar arrangement, while the slightly distorted octahedral coordination is completed by the two symmetry related N atoms of the NA ligands (N1 and N1') in the axial positions. The near equalities of the C1—O1 [1.2614 (14) Å] and C1—O2 [1.2600 (14) Å] bonds in the carboxylate group indicate a delocalized bonding arrangement, rather than localized single and double bonds. The Zn—O bond lengths are 2.1047 (8) Å (for benzoate oxygens) and 2.1446 (8) Å (for water oxygens), and the Zn—N bond length is 2.1253 (10) Å, close to standard values (Allen *et al.*, 1987). The Zn atom is displaced out of the mean-plane of the carboxylate group (O1/C1/O2) by -0.6114 (1) Å. The dihedral angle between the planar carboxylate group and the adjacent benzene ring A (C2—C7) is 24.13 (8)°. The benzene A (C2—C7) and the pyridine B (N1/C9—C13) rings are oriented at a dihedral angle of A/B = 88.52 (4)°. The coordinating water molecule links with the carboxylate group *via* an O—H···O hydrogen bond (Table 1).

In the crystal, N—H···O and O—H···O hydrogen bonds, and a weak C-H···π interaction (Table 1) link the molecules into a two-dimensional network parallel to plane (010). These networks are linked via C-H···O and π–π interactions [Cg1···Cg1^i^ = 3.8483 (7) Å; symmetry code: (i) 1 - x, 1 - y, 2 - z, where Cg1 is the centroid of ring A (C2—C7)] to form a three-dimensional supramolecular structure.

### Experimental

The title compound was prepared by the reaction of ZnSO_4_.H_2_O (0.90 g, 5 mmol) in H_2_O (25 ml) and NA (1.22 g, 50 mmol) in H_2_O (100 ml) with sodium 4-formylbenzoate (1.72 g, 10 mmol) in H_2_O (100 ml) at room temperature. The mixture was filtered and set aside to crystallize at ambient temperature for several days, giving colourless single crystals.

### Refinement

Atoms H8 (for CH), H21 and H22 (for NH_2_) and H51 and H52 (for H_2_O) were located in a difference Fourier map and were refined freely. The C-bound H-atoms were positioned geometrically and constrained to ride on their parent atoms: C—H = 0.93 Å with *U*_iso_(H) = 1.2*U*_eq_(C).

### Crystal data

**Table:**

[Zn(C_8_H_5_O_3_)_2_(C_6_H_6_N_2_O)_2_(H_2_O)_2_]	*Z* = 1
*M**_r_* = 643.92	*F*(000) = 332
Triclinic, *P*1	*D*_x_ = 1.528 Mg m^−^^3^
Hall symbol: -P 1	Mo *K*α radiation, λ = 0.71073 Å
*a* = 7.7861 (2) Å	Cell parameters from 9923 reflections
*b* = 9.7877 (3) Å	θ = 2.7–28.5°
*c* = 9.9087 (3) Å	µ = 0.94 mm^−^^1^
α = 77.851 (3)°	*T* = 100 K
β = 71.462 (2)°	Block, colourless
γ = 86.720 (3)°	0.44 × 0.37 × 0.20 mm
*V* = 699.87 (4) Å^3^	

### Data collection

**Table:**

Bruker Kappa APEXII CCD area-detector diffractometer	3475 independent reflections
Radiation source: fine-focus sealed tube	3413 reflections with *I* > 2σ(*I*)
Graphite monochromator	*R*_int_ = 0.019
φ and ω scans	θ_max_ = 28.5°, θ_min_ = 2.1°
Absorption correction: multi-scan (*SADABS*; Bruker, 2005)	*h* = −10→10
*T*_min_ = 0.682, *T*_max_ = 0.831	*k* = −13→13
12257 measured reflections	*l* = −11→13

### Refinement

**Table:**

Refinement on *F*^2^	Primary atom site location: structure-invariant direct methods
Least-squares matrix: full	Secondary atom site location: difference Fourier map
*R*[*F*^2^ > 2σ(*F*^2^)] = 0.022	Hydrogen site location: inferred from neighbouring sites
*wR*(*F*^2^) = 0.060	H atoms treated by a mixture of independent and constrained refinement
*S* = 1.06	*w* = 1/[σ^2^(*F*_o_^2^) + (0.0274*P*)^2^ + 0.3774*P*] where *P* = (*F*_o_^2^ + 2*F*_c_^2^)/3
3475 reflections	(Δ/σ)_max_ < 0.001
216 parameters	Δρ_max_ = 0.43 e Å^−^^3^
0 restraints	Δρ_min_ = −0.31 e Å^−^^3^

### Special details

**Table:**

Geometry. All esds (except the esd in the dihedral angle between two l.s. planes) are estimated using the full covariance matrix. The cell esds are taken into account individually in the estimation of esds in distances, angles and torsion angles; correlations between esds in cell parameters are only used when they are defined by crystal symmetry. An approximate (isotropic) treatment of cell esds is used for estimating esds involving l.s. planes.
Refinement. Refinement of F^2^ against ALL reflections. The weighted R-factor wR and goodness of fit S are based on F^2^, conventional R-factors R are based on F, with F set to zero for negative F^2^. The threshold expression of F^2^ > 2sigma(F^2^) is used only for calculating R-factors(gt) etc. and is not relevant to the choice of reflections for refinement. R-factors based on F^2^ are statistically about twice as large as those based on F, and R- factors based on ALL data will be even larger.

### Fractional atomic coordinates and isotropic or equivalent isotropic displacement parameters (Å^2^)

**Table:**

	*x*	*y*	*z*	*U*_iso_*/*U*_eq_	
Zn1	0.0000	0.0000	0.0000	0.01128 (6)	
O1	0.12362 (11)	0.18202 (9)	0.00926 (9)	0.01501 (16)	
O2	−0.11031 (11)	0.27782 (9)	0.15725 (9)	0.01613 (17)	
O3	0.49521 (14)	0.66936 (13)	0.32150 (15)	0.0404 (3)	
O4	−0.43332 (12)	0.00146 (9)	−0.33495 (9)	0.01797 (17)	
O5	0.26589 (11)	−0.08294 (9)	−0.07850 (10)	0.01534 (16)	
H51	0.345 (3)	−0.042 (2)	−0.144 (2)	0.033 (5)*	
H52	0.238 (3)	−0.151 (2)	−0.110 (2)	0.038 (5)*	
N1	−0.00282 (13)	0.09203 (10)	−0.21362 (11)	0.01310 (18)	
N2	−0.32692 (15)	0.13245 (12)	−0.56057 (11)	0.0178 (2)	
H21	−0.245 (2)	0.1865 (18)	−0.6267 (19)	0.025 (4)*	
H22	−0.411 (2)	0.1018 (19)	−0.584 (2)	0.027 (4)*	
C1	0.05609 (15)	0.26503 (11)	0.09352 (12)	0.0128 (2)	
C2	0.18554 (15)	0.35243 (11)	0.12616 (12)	0.0128 (2)	
C3	0.36219 (15)	0.30780 (12)	0.11172 (13)	0.0151 (2)	
H3	0.4044	0.2294	0.0723	0.018*	
C4	0.47558 (16)	0.38054 (12)	0.15623 (14)	0.0169 (2)	
H4	0.5932	0.3501	0.1479	0.020*	
C5	0.41325 (16)	0.49869 (12)	0.21317 (14)	0.0170 (2)	
C6	0.23814 (16)	0.54657 (13)	0.22349 (15)	0.0194 (2)	
H6	0.1980	0.6273	0.2590	0.023*	
C7	0.12464 (15)	0.47344 (12)	0.18077 (14)	0.0168 (2)	
H7	0.0075	0.5046	0.1883	0.020*	
C8	0.53693 (18)	0.57168 (15)	0.26194 (17)	0.0257 (3)	
H8	0.665 (2)	0.5325 (17)	0.2463 (18)	0.021 (4)*	
C9	−0.14410 (15)	0.06794 (11)	−0.25551 (12)	0.0132 (2)	
H9	−0.2391	0.0122	−0.1889	0.016*	
C10	−0.15524 (15)	0.12221 (11)	−0.39338 (12)	0.0131 (2)	
C11	−0.01372 (16)	0.20686 (13)	−0.49174 (13)	0.0178 (2)	
H11	−0.0168	0.2454	−0.5851	0.021*	
C12	0.13251 (16)	0.23309 (13)	−0.44870 (13)	0.0192 (2)	
H12	0.2284	0.2897	−0.5127	0.023*	
C13	0.13337 (15)	0.17388 (12)	−0.30955 (13)	0.0156 (2)	
H13	0.2316	0.1913	−0.2812	0.019*	
C14	−0.31742 (15)	0.08240 (12)	−0.42808 (12)	0.0140 (2)	

### Atomic displacement parameters (Å^2^)

**Table:**

	*U*^11^	*U*^22^	*U*^33^	*U*^12^	*U*^13^	*U*^23^
Zn1	0.01063 (9)	0.01353 (9)	0.01123 (9)	−0.00014 (6)	−0.00480 (6)	−0.00367 (6)
O1	0.0147 (4)	0.0165 (4)	0.0146 (4)	−0.0020 (3)	−0.0042 (3)	−0.0051 (3)
O2	0.0119 (4)	0.0183 (4)	0.0189 (4)	−0.0008 (3)	−0.0044 (3)	−0.0056 (3)
O3	0.0237 (5)	0.0457 (7)	0.0640 (8)	−0.0008 (5)	−0.0123 (5)	−0.0392 (6)
O4	0.0172 (4)	0.0235 (4)	0.0141 (4)	−0.0062 (3)	−0.0064 (3)	−0.0016 (3)
O5	0.0122 (4)	0.0179 (4)	0.0160 (4)	−0.0007 (3)	−0.0034 (3)	−0.0050 (3)
N1	0.0130 (4)	0.0147 (4)	0.0129 (4)	−0.0003 (3)	−0.0053 (3)	−0.0036 (3)
N2	0.0183 (5)	0.0233 (5)	0.0135 (5)	−0.0057 (4)	−0.0080 (4)	−0.0014 (4)
C1	0.0140 (5)	0.0127 (5)	0.0120 (5)	−0.0013 (4)	−0.0060 (4)	0.0002 (4)
C2	0.0128 (5)	0.0131 (5)	0.0125 (5)	−0.0018 (4)	−0.0042 (4)	−0.0018 (4)
C3	0.0141 (5)	0.0136 (5)	0.0184 (5)	0.0005 (4)	−0.0052 (4)	−0.0050 (4)
C4	0.0125 (5)	0.0170 (5)	0.0227 (6)	0.0010 (4)	−0.0068 (4)	−0.0054 (4)
C5	0.0141 (5)	0.0176 (5)	0.0207 (6)	−0.0026 (4)	−0.0053 (4)	−0.0066 (4)
C6	0.0159 (5)	0.0164 (5)	0.0274 (6)	0.0003 (4)	−0.0050 (5)	−0.0108 (5)
C7	0.0124 (5)	0.0162 (5)	0.0227 (6)	0.0011 (4)	−0.0055 (4)	−0.0062 (4)
C8	0.0166 (6)	0.0289 (7)	0.0367 (8)	−0.0025 (5)	−0.0086 (5)	−0.0162 (6)
C9	0.0127 (5)	0.0141 (5)	0.0138 (5)	−0.0011 (4)	−0.0048 (4)	−0.0033 (4)
C10	0.0135 (5)	0.0144 (5)	0.0128 (5)	−0.0001 (4)	−0.0052 (4)	−0.0041 (4)
C11	0.0190 (5)	0.0219 (5)	0.0118 (5)	−0.0034 (4)	−0.0052 (4)	−0.0005 (4)
C12	0.0154 (5)	0.0230 (6)	0.0170 (6)	−0.0064 (4)	−0.0032 (4)	−0.0007 (5)
C13	0.0129 (5)	0.0175 (5)	0.0175 (5)	−0.0015 (4)	−0.0055 (4)	−0.0043 (4)
C14	0.0145 (5)	0.0158 (5)	0.0140 (5)	0.0001 (4)	−0.0061 (4)	−0.0053 (4)

### Geometric parameters (Å, º)

**Table:**

Zn1—O1	2.1047 (8)	C3—H3	0.9300
Zn1—O1^i^	2.1047 (8)	C4—C3	1.3902 (16)
Zn1—O5	2.1446 (8)	C4—C5	1.3883 (16)
Zn1—O5^i^	2.1446 (8)	C4—H4	0.9300
Zn1—N1	2.1253 (10)	C5—C6	1.3955 (17)
Zn1—N1^i^	2.1253 (10)	C5—C8	1.4803 (17)
O1—C1	1.2614 (14)	C6—H6	0.9300
O2—C1	1.2600 (14)	C7—C6	1.3822 (16)
O3—C8	1.2032 (17)	C7—H7	0.9300
O4—C14	1.2402 (14)	C8—H8	1.022 (17)
O5—H51	0.79 (2)	C9—C10	1.3859 (16)
O5—H52	0.86 (2)	C9—H9	0.9300
N1—C9	1.3413 (14)	C10—C11	1.3900 (16)
N1—C13	1.3437 (14)	C10—C14	1.5011 (15)
N2—C14	1.3250 (16)	C11—C12	1.3901 (17)
N2—H21	0.861 (18)	C11—H11	0.9300
N2—H22	0.847 (19)	C12—H12	0.9300
C1—C2	1.5082 (15)	C13—C12	1.3806 (17)
C2—C3	1.3912 (15)	C13—H13	0.9300
C2—C7	1.4009 (15)		
			
O1—Zn1—O1^i^	180.00 (2)	C3—C4—H4	120.0
O1—Zn1—O5	88.01 (3)	C5—C4—C3	119.98 (11)
O1^i^—Zn1—O5	91.99 (3)	C5—C4—H4	120.0
O1—Zn1—O5^i^	91.99 (3)	C4—C5—C6	120.33 (11)
O1^i^—Zn1—O5^i^	88.01 (3)	C4—C5—C8	118.35 (11)
O1—Zn1—N1	89.84 (3)	C6—C5—C8	121.32 (11)
O1^i^—Zn1—N1	90.16 (3)	C5—C6—H6	120.1
O1—Zn1—N1^i^	90.16 (3)	C7—C6—C5	119.71 (11)
O1^i^—Zn1—N1^i^	89.84 (3)	C7—C6—H6	120.1
O5^i^—Zn1—O5	180.00 (5)	C2—C7—H7	119.9
N1—Zn1—O5	92.47 (3)	C6—C7—C2	120.18 (11)
N1^i^—Zn1—O5	87.53 (3)	C6—C7—H7	119.9
N1—Zn1—O5^i^	87.53 (3)	O3—C8—C5	124.61 (12)
N1^i^—Zn1—O5^i^	92.47 (3)	O3—C8—H8	119.0 (9)
N1^i^—Zn1—N1	180.00 (6)	C5—C8—H8	116.4 (9)
C1—O1—Zn1	126.47 (7)	N1—C9—C10	123.12 (10)
Zn1—O5—H51	123.5 (14)	N1—C9—H9	118.4
Zn1—O5—H52	98.4 (13)	C10—C9—H9	118.4
H52—O5—H51	105.2 (18)	C9—C10—C11	118.07 (10)
C9—N1—Zn1	119.49 (8)	C9—C10—C14	117.71 (10)
C9—N1—C13	118.35 (10)	C11—C10—C14	124.20 (10)
C13—N1—Zn1	122.16 (8)	C10—C11—C12	119.12 (11)
C14—N2—H21	123.1 (12)	C10—C11—H11	120.4
C14—N2—H22	117.9 (12)	C12—C11—H11	120.4
H22—N2—H21	118.6 (17)	C11—C12—H12	120.5
O1—C1—C2	117.40 (10)	C13—C12—C11	119.04 (11)
O2—C1—O1	125.67 (10)	C13—C12—H12	120.5
O2—C1—C2	116.89 (10)	N1—C13—C12	122.30 (11)
C3—C2—C1	119.98 (10)	N1—C13—H13	118.8
C3—C2—C7	119.82 (10)	C12—C13—H13	118.8
C7—C2—C1	120.05 (10)	O4—C14—N2	122.62 (11)
C2—C3—H3	120.0	O4—C14—C10	119.55 (10)
C4—C3—C2	119.93 (10)	N2—C14—C10	117.79 (10)
C4—C3—H3	120.0		
			
O5—Zn1—O1—C1	−152.50 (9)	C1—C2—C3—C4	−173.27 (11)
O5^i^—Zn1—O1—C1	27.50 (9)	C7—C2—C3—C4	2.26 (17)
N1—Zn1—O1—C1	115.03 (9)	C1—C2—C7—C6	173.98 (11)
N1^i^—Zn1—O1—C1	−64.97 (9)	C3—C2—C7—C6	−1.55 (18)
O1—Zn1—N1—C9	−143.10 (8)	C5—C4—C3—C2	−0.88 (18)
O1^i^—Zn1—N1—C9	36.90 (8)	C3—C4—C5—C6	−1.22 (19)
O1—Zn1—N1—C13	37.17 (9)	C3—C4—C5—C8	178.85 (12)
O1^i^—Zn1—N1—C13	−142.83 (9)	C4—C5—C6—C7	1.9 (2)
O5—Zn1—N1—C9	128.89 (8)	C8—C5—C6—C7	−178.14 (12)
O5^i^—Zn1—N1—C9	−51.11 (8)	C4—C5—C8—O3	−174.99 (15)
O5—Zn1—N1—C13	−50.84 (9)	C6—C5—C8—O3	5.1 (2)
O5^i^—Zn1—N1—C13	129.16 (9)	C2—C7—C6—C5	−0.54 (19)
Zn1—O1—C1—O2	−21.18 (16)	N1—C9—C10—C11	−0.77 (17)
Zn1—O1—C1—C2	156.07 (7)	N1—C9—C10—C14	177.58 (10)
Zn1—N1—C9—C10	−179.02 (8)	C9—C10—C11—C12	0.27 (17)
C13—N1—C9—C10	0.72 (17)	C14—C10—C11—C12	−177.96 (11)
Zn1—N1—C13—C12	179.57 (9)	C9—C10—C14—O4	−1.27 (16)
C9—N1—C13—C12	−0.16 (17)	C9—C10—C14—N2	−178.98 (10)
O1—C1—C2—C3	−23.65 (15)	C11—C10—C14—O4	176.97 (11)
O1—C1—C2—C7	160.83 (11)	C11—C10—C14—N2	−0.74 (17)
O2—C1—C2—C3	153.83 (11)	C10—C11—C12—C13	0.23 (19)
O2—C1—C2—C7	−21.68 (16)	N1—C13—C12—C11	−0.30 (19)

Symmetry code: (i) −*x*, −*y*, −*z*.

### Hydrogen-bond geometry (Å, º)

Cg2 is the centroid of the pyridine ring.

**Table:**

*D*—H···*A*	*D*—H	H···*A*	*D*···*A*	*D*—H···*A*
N2—H21···O2^ii^	0.862 (17)	2.087 (17)	2.8789 (13)	152.4 (16)
N2—H22···O4^iii^	0.849 (17)	2.058 (18)	2.8904 (15)	166.4 (18)
O5—H51···O4^iv^	0.79 (2)	2.10 (2)	2.8597 (13)	161 (2)
O5—H52···O2^i^	0.86 (2)	1.85 (2)	2.6845 (13)	163 (2)
C4—H4···O2^iv^	0.93	2.40	3.3245 (16)	173
C13—H13···O3^v^	0.93	2.47	3.3083 (17)	150
C6—H6···*Cg*2^vi^	0.93	2.72	3.6361 (14)	167

Symmetry codes: (i) −*x*, −*y*, −*z*; (ii) *x*, *y*, *z*−1; (iii) −*x*−1, −*y*, −*z*−1; (iv) *x*+1, *y*, *z*; (v) −*x*+1, −*y*+1, −*z*; (vi) −*x*, −*y*+1, −*z*+2.
